# Diversity and utilization of antimalarial ethnophytotherapeutic remedies among the Kikuyus (Central Kenya)

**DOI:** 10.1186/1746-4269-2-8

**Published:** 2006-02-01

**Authors:** Grace N Njoroge, Rainer W Bussmann

**Affiliations:** 1 Jomo Kenyatta University of Agriculture Science and Technology, Botany Department, P.O. Box 62000, Nairobi, Kenya; 2 Harold L. Lyon Arboretum, University of Hawaii, 3860 Manoa Rd., Honolulu, HI 96822, USA

## Abstract

Plants in Kenya are becoming increasingly important as sources of traditional medicines. The World Health Organization (WHO) has estimated that malaria kills about 2.7 million people every year, 90% of who are from Africa. Malaria continues to be a national concern in Kenya as it plays a major role in the high mortality rates being experienced currently. The use and miss-use of chloroquine to prevent and treat falciparium malaria has led to widespread appearance of chloroquine resistant parasites in Kenya and other tropical countries. These factors and the rising costs of non-chloroquine drugs have made the local people to turn to traditional remedies for management of this menace.

This paper examines the current utilization of traditional plant medicines in managing malaria menace in Central Kenya. The results show both indigenous and introduced species are in use indicating traditional medicinal practices in this region are dynamic. In total 58 species in 54 genera and 33 families were identified. The family Rubiaceae was found to have the highest number of reported species. Use of the various taxa is compared between five districts within Central Province of Kenya. The commonest species in this pharmacopoeia are: *Caesalpinia volkensii *Harms, *Strychnos henningsii *Gilg, *Ajuga remota *Benth., *Warbugia ugandensis *Sprague and *Olea europaea *L. The first three species are used in all the five districts while the others are restricted in some of the districts. In 74% of the anti-malarial plant species reported in this study, the remedies are obtained in destructive manner and may need conservation measures to ensure sustainable utilization. The results of this study become a basis for selecting plants for further pharmacological and phytochemical studies in developing new and locally relevant anti-malarial agents.

## Background

Malaria has continued to be a major global public health problem and a health concern in most African countries. It is thought that malaria is by far the most serious tropical disease causing one to two million deaths per year in Africa [1,2]. The World Health Organization (WHO) has estimated that about 2 billion people in over 100 countries are exposed to malaria [2]. The worsening economic situation of the Sub-Sahara African countries makes it difficult to expand modern health services hence effective low-cost delivery medical system is urgently needed [3].

In Kenya, malaria continues to be a national concern as it plays a major role in the high mortality seen in infants and children. It is also responsible for abortion, premature deliveries, growth retardation, low birth weight and anemia [4-7]. In Kenya malaria is responsible for 30–50% out patient treatments, 19% admissions and accounts for 8–10 million treatments per year [8]. *Anopheles gambiae *s.l. and *Anopheles funestus *are the primary vectors of malaria in East Africa [9].

The use and miss-use of chloroquine to prevent and treat *falciparium *malaria has led to widespread appearance of chloroquine resistant parasites in Kenya and other tropical countries [2]. Kenya has a diverse climate and ecology, which leads to a wide variation in malaria and subsequent disease epidemiology [8]. This is complicated by the fact that global warming may lead to expansion of areas in which the ambient temperature and climatic conditions are suitable for Plasmodium transmission [10]. Since 1988, malaria epidemics have occurred in multiple sites in Kenya highlands. Climatic variability has been associated with some of the recent epidemics [6].

Kenya, through the division of malaria control (DOMC), ministry of health has developed several strategies for dealing with malaria. In regard to highland malaria for example, management strategies have been proposed which include, improved planning for the annual resurgent outbreak, augmented by simple central nationwide early warning which is likely to lead to increased epidemic preparedness [7]. Other key strategic approaches to malaria control in Kenya include, case management, providing malaria prevention and control to pregnant women, ensuring use of insecticide treated nets (ITNs) as well as improving malaria preparedness and response [11].

Despite these efforts the disease is still rampant, with over 170 million working days lost annually and treatment unaffordable to many Kenyans [8]. Factors that make malaria treatment unaffordable include, rising costs of non-chloroquine drugs, high poverty levels as well high prices of insecticide treated nets [12].

Some of the plants used in malaria control in Kenya are used as repellants for the mosquitoes [13] while others are taken, as medicines [14]. The drug resistant phenomenon has created urgent need to search for new drugs and alternative medicines for malaria and other diseases in Kenya [15]. Some of the plants used traditionally for malaria treatment have been investigated for their efficacy with positive results. The extracts tested in vitro have been shown to be active against chloroquine (CQ)- sensitive and resistant strains of *Plasmodium falciparum*. Such plant extracts have been recommended for use as sources for novel anti-malarial compounds to be used alone or in combination with chloroquin [14]. The plants tested are far too few. An often-limiting factor to these investigations is lack of comprehensive ethnobotanical data to help choose plant candidates for potency/efficacy tests.

Some ethnobotanical studies have been accomplished in Kenya targeting the different people groups/tribes and localities [16-26] among others. These studies cover various aspects of plant utilisation by local communities in Kenya. Studies however, on anti-malarial plants targeting the Kikuyus have not been done.

Effort was made in this study to indicate the frequency of mention of each anti-malarial plant species in the entire survey as an estimation of agreement on use in this region. The results provide data for further pharmacological and phytochemical studies. Since the plant parts utilized in preparation of anti-malarial remedies are reported in this survey, it serves as an indication of species that may need further ecological assessment on their regeneration status.

## Methodology

### Subjects and study area

**Figure 1:**
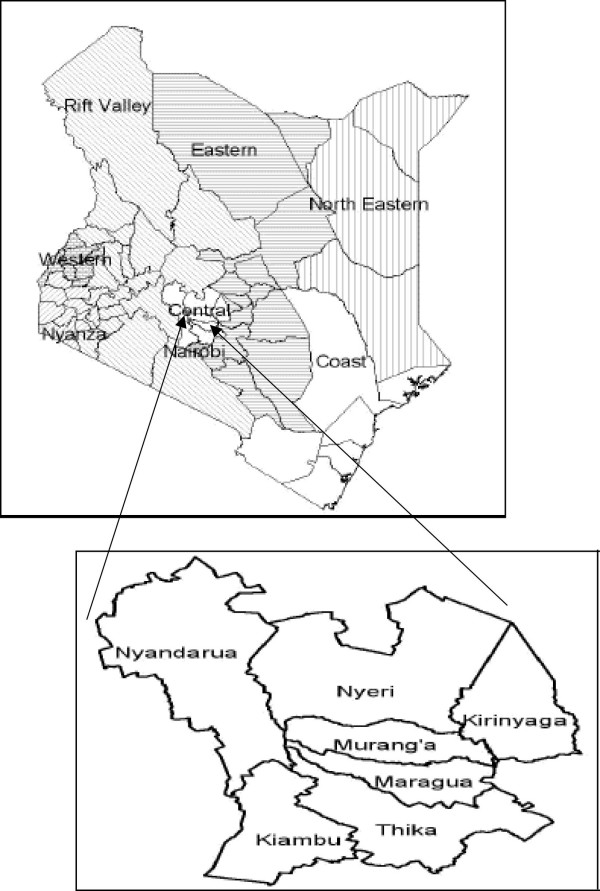
Map of Kenya with main provinces and all districts in Central Kenya where the Kikuyu mainly live.

The Kikuyu people mostly occupy the Central Province of Kenya (Fig. 1), which is often referred to as the Kikuyu escarpment. Half of the 2.9 million Kenyans living within 5 km of the forest are in Central Kenya [27]. From the latest census (1998) the province has a population of about 3724159 people, of which, 1828616 are males and 1895543 are female. Farming is the main economic activity in the area with coffee and tea as the main cash crops.

This region has high population density and large concentration of forests, which are facing intense pressure due to over-utilization and hence medicinal plants may be disappearing before their uses are documented. Care was taken in this study however, to collect plants mentioned as useful anti-malarial remedies outside the forests. This is especially because non-forest species such as weeds are now known to be important sources of pharmaceuticals [28].

### Data collection and analysis

Fieldwork was carried out between January, 2001- October, 2004 as part of a larger ethnobotanical work covering this region. To obtain the plants used traditionally for management of malaria, interviews were conducted using semi-structured questionnaires. Random sampling technique was applied in distributing the questionnaires. Before carrying out the interviews however, an oral consent was sought from every respondent.

A total of 59 respondents were interviewed, these included males and females that depended on wild plants as sources of anti-malarial medicines either for self-medication or for treating others among the Kikuyus. Prior to surveys in each area, a research assistant was identified who had grown up in the area and knew the people and the language well. The assistant accompanied the researcher during the interviews and was helpful in wining trust of the respondents as well as in explaining any hidden meanings. The Kikuyu word for malaria is almost the same as the English word and therefore it was easy to communicate to the local people the nature of the disease remedy under investigation. In some cases malaria was equated to fever and joint pains, plant records for such are not included in this study as they may not necessarily be anti-malaria remedies.

In the cases where the local people recommended specific persons as excellent in knowledge of traditional herbal medicines, such respondents accompanied the researcher to collect ethnobotanical specimens [29]. During such missions personal discussions on management of malaria through herbal preparations as well as local means of identifying anti-malarial plants were encouraged.

Various ethnographic skills were applied in the fieldwork such as direct observations of what the local people were using or selling in the markets, taking part in the local peoples activities such as weeding, medicinal plants harvests and gathering of wild fodder. Group interviews were sometimes carried out especially where there was need to identify most preferred anti-malarial herbal remedies in the study area [30].

Fieldwork for this study concentrated on five main districts in Central Kenya: Kiambu, Maragwa, Murang'a, Nyeri and Thika. This was a purposeful sampling of the districts as the other two districts not sampled (Nyandarua and Kirinyaga) were found to be uniquely different from these five. Nyandarua district, for example was formally an area occupied by white settlers and has only recently been occupied by local communities when the land was sub-divided. Kirinyaga district comprises of a group of Kikuyu people with a different dialect and are likely to have different indigenous knowledge form the other districts.

Data on plant species and families, parts used, plant habit, the vernacular name and district were entered in excel worksheets and frequency of each species worked out (Table 2). Voucher specimens were collected and are preserved at the Jomo Kenyatta University herbarium, Botany department while duplicates will be sent to the East African Herbarium (EA). Plant identifications were done partly by the first author and partly by Mr. Simeon Mathenge, a botanist at the university of Nairobi Herbarium.

## Results

In total 58 species in 54 genera and 33 families were identified as anti-malarial herbal remedies in this region (Table 2). The commonest species in this pharmacopoeia are: *Caesalpinia volkensii, Strychnos henningsii, Ajuga remota, Warbugia ugandensis *and *Olea europaea*. The first three species are used in all the five districts while the others are restricted in some of the districts.

Based on frequency with which the respondents mentioned the anti-malarial species, it was possible to establish the five most important species for each district (Table 1).

**Table 1:** Most frequently mentioned plant species in management of malaria by district in Central Kenya.

**Kiambu District**	**Frequency**	**Maragwa District**	**Frequency**	**Murang'a District**	**Frequency**	**Nyeri District**	**Frequency**	**Thika District**	**Frequency**
*Caesalpinia volkensii*	7	*Caesalpinia volkensii*	12	*Caesalpinia volkensii*	4	*Olea europeaea*	6	*Ajuga remota*	5
*Rhoicissus tridentata*	5	*Fagaropsis angolensis*	7	*Strychnos henningsii*	4	*Ajuga remota*	5	*Caesalpinia volkensii*	5
*Aloe kedongensis*	4	*Strychnos henningsii*	6	*Aloe lateritia*	4	*Strychnos henningsii*	5	*Strychnos henningsii*	3
*Ajuga remota*	3	*Pentas longiflora*	5	*Ajuga remota*	4	*Rhamnus prinoides*	3	*Trimeria grandifolia*	2
*Senna didymobotrya*	3	*Warburgia ugandensis*	5	*Zanthoxylum chalybeum*	2	*Azardirachta indica*	3	*Warburgia ugandensis*	2

**Table 2:** List Of Species Mentioned In This Study As Used For Malaria Management By Frequency Of Mention In Central Kenya

Local name (Kikuyu)	Latin Name	Family Name	Number of times mentioned	Place cited
mubuthi/mucuthi	*Caesalpinia volkensii *Harms	Caesalpiniaceae	37	Kiambu, Maragwa, Muranga, Nyeri & Thika
muteta	*Strychnos henningsii *Gilg	Loganiaceae	26	Thika, Nyeri, Murang'a, Maragwa, Kiambu
wanjiru	*Ajuga remota *Benth	Lamiaceae	18	Kiambu, Muranga, Nyeri, Thika
Muthiga	*Warburgia ugandensis *Sprague	Canellaceae	11	Kiambu, Maragwa, Nyeri & Thika
Mutamaiyu	*Olea europaea *L.	Oleaceae	10	Kiambu, Maragwa & Nyeri
mukarakinga	*Rahmnus prinoides *L. He'rit	Rhamnaceae	8	Nyeri, Maragwa & Kiambu
mukaragati	*Fagaropsis angolensis *(Engl.) Dale	Rutaceae	7	Maragwa
mukenera	*Zanthoxylum chalybeum *Engl.	Rutaceae	7	Maragwa, Murang'a & Thika
mwinu	*Senna didymobotrya*(Fresen.) Irwin & Barneby	Caesalpinaceae	6	Kiambu, Maragwa & Nyeri
mugwanugu	*Aloe kedongensis *Reynolds	Liliaceae	6	Kiambu & Thika
mwarubaine	*Azadirachta indica *A.Juss.	Meliaceae	6	Kiambuu, Murang'a & Nyeri
Muthura	*Rhus vulgaris *Meikle	Anacardiaceae	5	Maragwa
Muhindahindi	*Trimeria grandifolia *(Hochst.) Warb.	Flacourtiaceae	5	Thika, Nyeir & maragwa
kiiruma	*Aloe lateritia *Engl.	Liliaceae	5	Maragwa & Murang'a
Muhuti	*Erythrina abyssinica *DC	Papilionaceae	5	Maragwa
Muhuha	*Pentas longiflora *Oliv.	Rubiaceae	5	Maragwa
Mutimu	*Citrus aurantiifolia *(Christm.) Swingle	Rutaceae	5	Maragwa
Munderendu	*Teclea simplicifolia *(Engl.) Verdoorn	Rutaceae	5	Maragwa
Munjuga Iria	*Clerodendrum myricoides *(Hochst.) Vatke	Verbenaceae	5	Maragwa & Nyeri
ndurutua	*Rhoicissus tridentata *(L.f.) Wild & Drummond	Vitaceae	5	Kiambu
Ruithiki/Mukenia/ Ruithiki	*Lantana camara *L.	Verbenaceae	4	Murang'a & maragwa
Mukawa	*Carissa edulis *(Forssk.)Vahl	Apocynaceae	3	Maragwa & Murang'a
Gakuinini	*Schkuhria pinnata *(Lam.) Thell.	Asteraceae	3	Thika & maragwa
muthuthi	*Maytenus senegalensis *(Lam.) Exell.	Celastraceae	3	Maragwa
muthima mburi	*Clutia abyssinica *Jaub. & Spach	Euphorbiaceae	3	Maragwa
Maruru	*Tithonia diversifolia *(Hemsl.) Gray	Asteraceae	2	Thika & Maragwa
gakungui	*Cucumis aculeatus *Cogn.	Cucurbitaceae	2	Kiambu
gacuki	*Ocimum kilimandscharicum *Guaerke	Lamiaceae	2	Murang'a
muthaiti	*Ocotea usambarensis *Engl.	Lauraceae	2	Maragwa & Nyeri
mubau	*Eucalyptus globulus *Labil.	Myrtaceae	2	Maragwa & Nyeri
Mwikunya	*Scutia myrtina *(Burm.f.) Harms	Rhamnaceae	2	Maragwa
muthathi	*Cassipourea malosana *(Bak.) Alston	Rhizophoraceae	2	Thika
Muiri	*Prunus africana *(Hook. f.) Kalkm.	Rosaceae	2	& Thika
mururue	*Toddalia asiatica *(L.) Lam.	Rutaceae	2	Nyeri & maragwa
muthithii	*Osyris lanceolata *Hochst. & Steudel	Santalaceae	2	Kiambu
thabai	*Urtica massaica *Mildbr.	Urticaceae	2	Kiambu
muthigiu	*Rhus natalensis *Krauss	Anacardiaceae	1	Murang'a
Muimbathunu	*Periploca linearifolia *Dill. & A. Rich.	Asclepiadaceae	1	Thika
mukungugu	*Commiphora eminii *Engl.	Burseraceae	1	Murang'a
Mahuithia	*Kalanchoe lanceolata *(Forsk.) Pers.	Crassulaceae	1	Thika
mukinduri	*Croton megalocarpus *Del.	Euphorbiaceae	1	Maragwa
kariria	*Euphorbia tirucalli *L.	Euphorbiaceae	1	Maragwa
kaiyaba	*Dovyalis caffra *(Hook. F. & Herv.) Warb	Flacourtiaceae	1	Kiambu
mukambura	*Dovyalis abyssinica *A. Rich	Flacourtiaceae	1	Maragwa
muthigira	*Hydnora abyssinica *Schweinf.	Hydnoraceae	1	Murang'a
mutoo	*Azanza gackeana *(F.Hoffm.) Excell & Hillcoat	Malvaceae	1	Maragwa
mukuriahungu	*Ekebergia capensis *Sparrm.	Meliaceae	1	Maragwa
Mucugu	*Cajanus cajan *Millsp.	Papilionaceae	1	Maragwa
Mwaritha	*Dalbergia lactea *Vatke	Papilionaceae	1	Thika
hondo	*Passiflora ligularis *A.Juss.	Passifloraceae	1	Kiambu
munyamati	*Pittosporum lanatum *Hutch. & Bruce	Pittosporaceae	1	Nyeri
ngukura	*Rhamnus staddo *A. Rich	Rhamnaceae	1	Nyeri
Mugukuma	*Keetia gueinzii *(Sond.) Bridson	Rubiaceae	1	Maragwa
mugucwa	*Zanthoxylum usambarense *(Engl.) Kokwaro	Rutaceae	1	Maragwa
Muba	*Pappea capensis *(Spreng) Eckl. & Zeyh.	Sapidiaceae	1	Maragwa
murumbae	*Withania somnifera *(L.) Dunal	Soalanaceae	1	Maragwa
mutura	*Solanum aculeastrum *Dunal	Solanaceae	1	Maragwa
mutambi	*Cyphostemma maranguense *(Gilg) Desc.	Vitaceae	1	Maragwa

**Figure 2:**
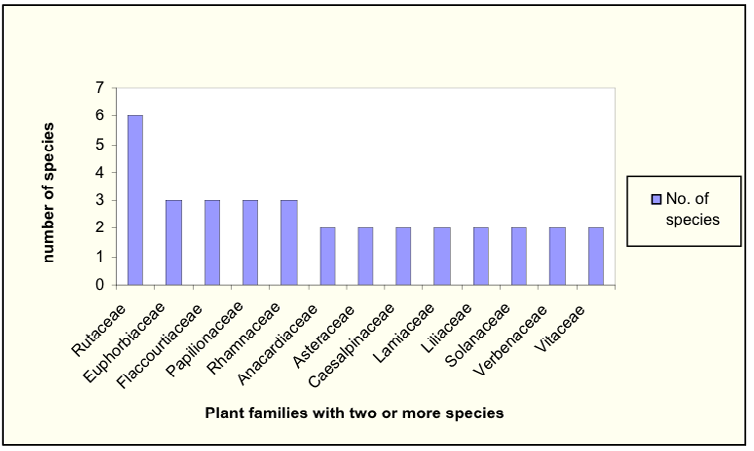
Plant families with two or more species used in malaria treatment in Central Kenya.

Of the families mentioned in this study the Rubiaceae had the highest number of species used in the treatment of malaria in Central Kenya. Thirteen families had at least two species mentioned as important in malaria treatment (Fig. 2).

**Figure 3:**
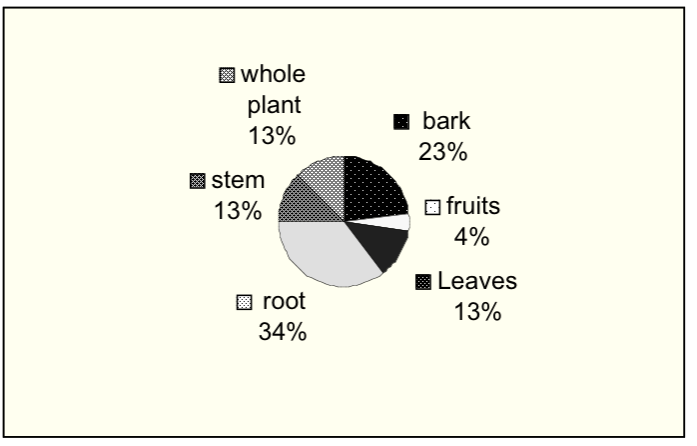
Plant parts utilized in management of malaria (Central Kenya).

**Figure 4:**
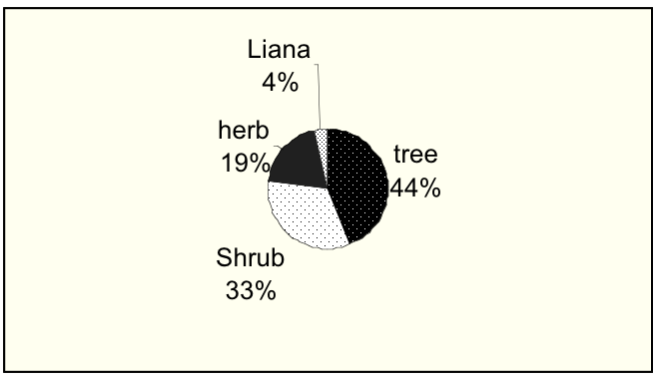
Habit of the plant species used in management of malaria in Central Kenya.

Various parts of the plants were utilized in preparation of anti-malarial herbal remedies in this area. In majority of the species (34%) the medicines were obtained from the roots (Fig. 3). Except for plants where the drugs are obtained from leaves the use of fruits, bark or uprooting the whole plant of a given species were found to be destructive means of obtaining the anti-malarial herbal remedies. Results from the habit of anti-malarial species shows that 77% of the anti-malarial herbal remedies are obtained from trees and shrubs (Fig. 4).

**Figure 5:**
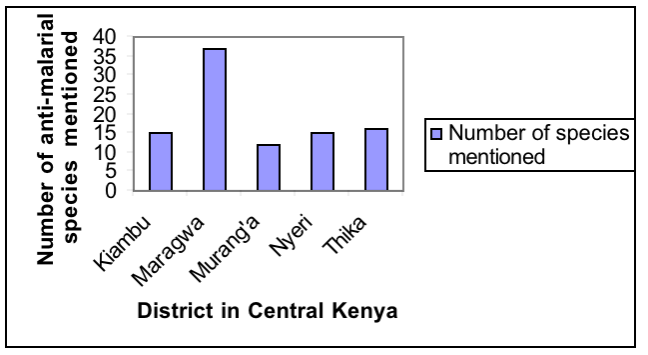
Number of anti-malarial plant species mentioned per district in Central Kenya.

The number of cited anti-malarial plant species varied between districts (Fig. 5). The highest number of plant species was cited in Maragwa district while the other districts had almost equal numbers.

Maragwa is classified as one of the districts with endemic malaria whiles the other districts may not have much malaria transmission throughout the year. People in Maragwa unlike the other districts may be more knowledgeable about the anti-malarial plant species. Since this is a major health challenge in the area using local plant resources may be one way this people are dealing with the problem.

## Discussion and Conclusion

Malaria continues to be a major health challenge in Kenya especially due to resistance of Plasmodium to the drugs in use currently. The results of this study show both indigenous and introduced species are in use for malaria treatment. This may indicate that traditional medicinal practices in this region are dynamic. The information on frequently utilized anti-malarial plant specie (Table 2) is an important lead to the species that can be targeted for further phytochemical analysis. Since there is no safer, effective and cheaper anti-malarial remedies than chloroquine [31] in the treatment of malaria, development of new anti-malarial drugs especially from plant sources may be the way forward in dealing with global drug-resistant problems of malaria.

There are species, which were commonly cited in this study that are also known to be used as sources of anti-malarial remedies in other parts of Africa. These included: *Azadirachta indica, Eucalyptus globulus, Ocimum gratissimum*, *Azanza gackeana *and *Warburgia ugandensis*. [32] Other studies have shown that active substances have been obtained against Plasmodium sp., for example, o-Naphthoquinones have been isolated from *Azanza gackeana *with demonstratable anti-malarial activity [4]. Consequently, further studies could investigate anti-malarial activity of the active substances likely to be isolated from the plant species identified form the current study.

Some of the species cited in this study are also used for management of Malaria outside the African continent, these include *Eucalyptus globulus *and *Citrus aurantiifolia*, which are popularly used anti-malarial plants in Brazil [2]. This study revealed however, some very popular anti-malarial plant species in this region that may not be popular in other regions. These include: *Strychnos henningsii, Caesalpinia volkensii*, *Senna didymobotrya, Fagaropsis angolensis, Zanthoxylum chalybeum, Rahmnus prinoides, Olea europaea, Aloe kedongensis, Trimeria grandifolia, Teclea simplicifolia, Rhus vulgaris, Rhoicissus tridentata, Pentas longiflora *and *Ajuga remota*

Studies from other regions of Africa indicate the family Rubiaceae to have many species used in malaria management in different countries [33]. The current study however, has revealed other hitherto undocumented anti-malarial species that could be new records for this ailment.

The parts utilized show that most of the anti-malarial drugs (74%) are obtained from fruits, barks, roots and sometimes the whole plant is uprooted and used in the preparation of the drugs. This calls for conservation measures to facilitate sustainable utilization of these plant resources. Some of the anti-malarial tree species for example, *Warburgia ugandensis *are already known to be over-exploited and in some parts of Kenya rare [34].

Investigations into plants in the region with repellent abilities or fatal effects on mosquitoes need to be investigated for development of drugs that can eradicate or minimize these malaria vectors. Investigation of anti-malarial remedies in the districts of Central province that were not covered in this study is recommended. Ecological studies on regeneration of these plant species may form an interesting study which could provide data on management of these species for sustainable utilization.

The local community of Central Kenya is the owner of the traditional knowledge presented in this paper, consequently any benefits that may accrue from the use this knowledge must be shared with them.
